# Microfluidic Biosensors for Point-of-Care Nucleic Acid Amplification Tests

**DOI:** 10.3390/bios13010005

**Published:** 2022-12-21

**Authors:** Kieu The Loan Trinh

**Affiliations:** Department of Industrial Environmental Engineering, Gachon University, Seongnam-si 13120, Republic of Korea; trinhloan@gachon.ac.kr

This Special Issue of *Biosensors*, “Microfluidic Biosensors for Point-of-Care Nucleic Acid Amplification Tests” aims to gather original research papers and comprehensive reviews detailing novel research, fabrication methods, and applications, as well as the challenges and prospects of developing microfluidics for improved biosensing and diagnostics. We hope to attract the attention of researchers from various disciplines of biology, chemistry, medicine, and materials sciences, to accelerate progress in the field and encourage creative minds to develop novel research.

Nucleic acid amplification tests are effective tools for rapid and accurate diagnosis in healthcare, food safety, and environmental monitoring [1,2,3]. These methods identify specific DNA or RNA sequences of the target for detection with high specificity and sensitivity. This method provides rapid results. Due to their advantages, nucleic acid amplification tests have become commonplace in many settings. Nucleic acid amplification tests can be classified as conventional polymerase chain reaction (PCR) and isothermal nucleic acid amplification. PCR is amplification reaction based on the activity of DNA polymerase and reverse and forward primers, which is driven by thermal cycles. The thermal cycles assist the denaturation of the double strand, annealing of the primers, and the extension and production of a new strand. Isothermal nucleic acid amplification methods are alternative forms of PCR. The method requires only one constant temperature for nucleic acid amplification, eliminating the need for thermal cycles [4]. These methods consume less time than PCR without the need for bulky equipment such as thermocyclers. There are many kinds of isothermal amplification techniques, including loop-mediated isothermal amplification, recombinase polymerase amplification, and helicase-dependent amplification.

A microfluidic chip is a small reactor that can perform an analytical assay by confining and controlling the fluid inside the chip channel. Microfluidics offer many advantages: the ability to use small amounts of sample and reagents; low cost; and short analysis time [5,6,7]. The technology is ideal for simplifying complicated analysis process in one tiny device. Microfluidics have been intensively developed for diagnostic purposes, offering a rapid, accurate, sensitive, cheap, and automated platform for nucleic acid amplification tests.

Point-of-care testing (POCT) is laboratory analysis conducted near the site of the patient [8,9]. POCT can improve patient care by providing a rapid testing process without the need for bulky equipment [10,11]. POCT offers rapid tests within minutes and utilizes cheap and user-friendly devices [12,13,14]. The combination of microfluidics and POCT can increase the efficiency of detection and user accessibility, simplify the analysis process, shorten assay time, and improve accuracy and sensitivity [15,16,17]. 

This Special Issue aims to introduce the research progress of microfluidic biosensors for point-of-care nucleic acid amplification tests. Sun et al. proposed a sensitive electrochemical biosensor for rapid screening of the tumor biomarker TP53 gene mutation hotspot [18]. The device detects a target using self-assembly sulfhydryl ended hairpin DNA probes tagged with methylene blue onto a gold electrode. Upon recognizing target DNA, the probes changed their structure and produced electrochemical signal differences via differential pulse voltammetry. The sensor achieved a limit of detection as low as 10 nmol/L, with good specific recognition ability for single-nucleotide polymorphism (SNP). 

An all-dielectric metasurface biosensor for rapid detection of attomolar SAR-CoV-2 nucleic acids was proposed by Iwanaga [19]. In the study, nucleic acid amplification was combined with a metasurface biosensor which produced high fluorescence efficiency. The biosensor is composed of a microfluidic transparent chip and a pair of all-dielectric metasurfaces. The device can rapidly and sensitively detect SAR-CoV-2 in 1 h with the limit of detection of 100 RNA copies/140 µL. 

This Special Issue contains meaningful contributions from researchers using cutting-edge biosensing technologies for POCT applications to achieve rapid nucleic acid analysis, including electrochemical biosensing and all-dielectric metasurface biosensing. Together with microfluidic techniques, we expect these proposed schemes to advance patient wellness by ensuring faster diagnosis and better prognosis and bridging the gap between healthcare providers and patients. 

## Data Availability

Not applicable.
